# Supplementary material for: The Ability of Chlorophyll to Trap Carcinogen Aflatoxin B1: A Theoretical Approach
Source: Int J Mol Sci. 2022 May 28;23(11):6068. doi: 10.3390/ijms23116068 (PMC9181093; doi:10.3390/ijms23116068)
Supplement: Supplementary file 1 [file ijms-23-06068-s001.zip › ijms-1707298-supplementary.pdf]

The Ability of Chlorophyll to Trap Carcinogen Aflatoxin B_1, a Theoretical Approach.

Alma Vázquez-Durán, Guillermo Téllez-Isaías, Maricarmen Hernández-Rodríguez, René Miranda Ruvalcaba, Joel Martínez, María Inés Nicolás-Vázquez, Juan Manuel Aceves-Hernández, Abraham Méndez-Albores

Content

Figure S1. M06-2X/6-311G(d,p) optimized geometries of AFB_1. Atomic charges of selected atoms are in electron units.
Figure S2. Reactive sites attained in the electrostatic potential map. The total electron density isosurface mapped with the molecular electrostatic potential of AFB_1, **chl a 1**, and **chl a 2**. The density = 0.0004 and isovalue = 0.02, at a level of calculation M06-2X/6-311G(d,p). The color scheme for the MEP is as follows: blue for electron-deficient, partially positive charge; light blue for the slightly electron-deficient region; yellow for a slightly electron-rich region and red for electron-rich, partial negative charge [1].
Figure S3. HOMO (eV), LUMO (eV) and the inter-frontier molecular orbital energy gaps of the target molecules.
Figure S4. Optimized geometry: a) **chl a 1**-α-D-AFB_1, b) **chl a 1**-α-E-AFB_1, c) **chl a 1**-β-D-AFB_1, and d) **chl a 1**-β-E-AFB_1.
Figure S5. Optimized geometry: a) **chl a 2**-α-D-AFB_1, b) **chl a 2**-α-E-AFB_1, c) **chl a 2**-β-E-AFB_1, and d) **chl a 2**-β-D-AFB_1.
Figure S6. Optimized geometry of a) **chl a 1**-E-2AFB_1, and b) **chl a 1**-D-2AFB_1.
Figure S7. Optimized geometry of a) **chl a 2**-E-2AFB_1, and b) **chl a 2**-D-2AFB_1.
Figure S8. a) RMSD, and b) radius of gyration of **chl a 2**, obtained by MD simulation.

Table S1. Natural atomic charges (e^-) for **chl a 1**, **chl a 2**, AFB_1 and their complexes.
Table S2. Interaction energy, in kcal/mol, of complexes **chl a 1**-AFB_1, and **chl a 2**-AFB_1 in the gas phase.
Table S3. Interaction energy, in kcal/mol, of complexes **chl a 1**-2AFB_1, and **chl a 2**-2AFB_1 in water as solvent.
Table S4. Cartesian coordinates for the more stable optimized geometries.

AFB_1 = aflatoxin B_1
chl a 2 = folded chlorophyll a.
chl a 1-α-E-AFB_1 = **AFB_1** coordinated to unfolded chlorophyll a.
chl a 2-β-E-AFB_1 = **AFB_1** coordinated to folded chlorophyll a.
chl a 1-D-2AFB_1 = **2AFB_1** coordinate to unfolded chlorophyll a.
chl a 2-E-2AFB_1 = **2AFB_1** coordinate to folded chlorophyll a.
chl a 2-AFB_1c = an intermolecular hydrogen bond between **AFB_1** and chlorophyll a.

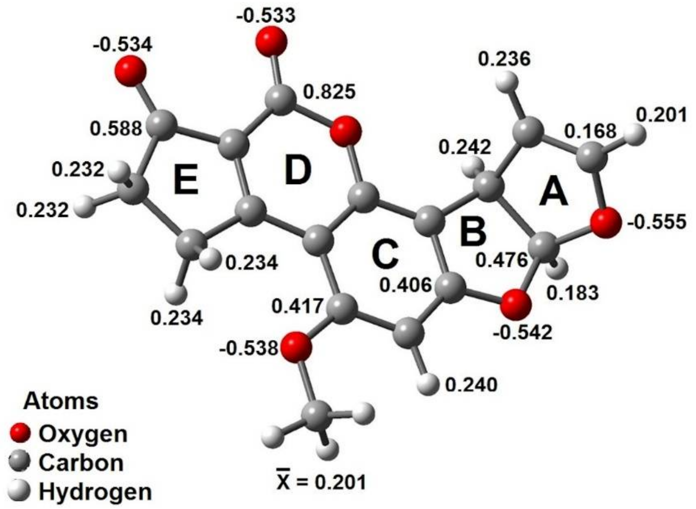

Figure S1. M06-2X/6-311G(d,p) optimized geometries of AFB_1. Atomic charges of selected atoms are in electron units.

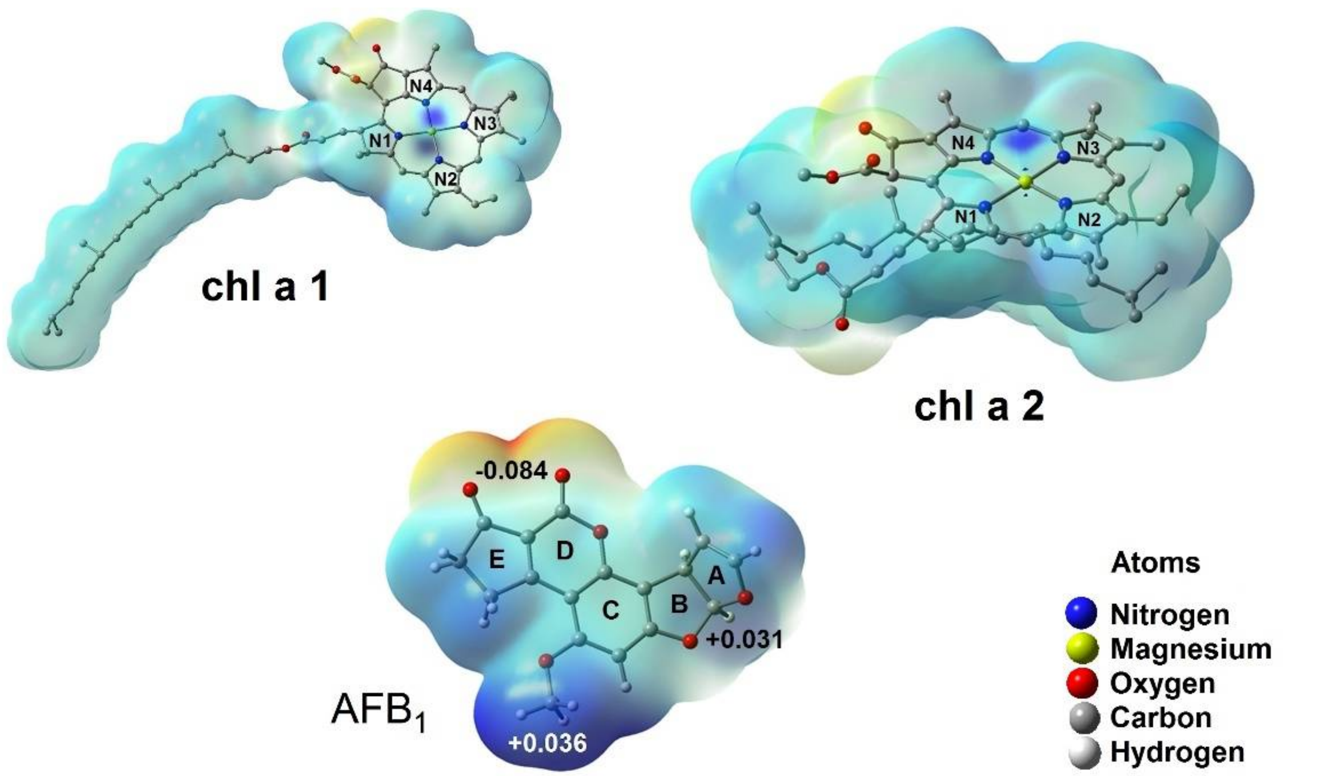

Figure S2. Reactive sites attained in the electrostatic potential map. The total electron density isosurface mapped with the molecular electrostatic potential of AFB_1, **chl a 1**, and **chl a 2**. The density = 0.0004 and isovalue = 0.02, at a level of calculation M06-2X/6-311G(d,p). The color scheme for the MEP is as follows: blue for electron-deficient, partially positive charge; light blue for the slightly electron-deficient region; yellow for a slightly electron-rich region and red for electron-rich, partial negative charge [1].

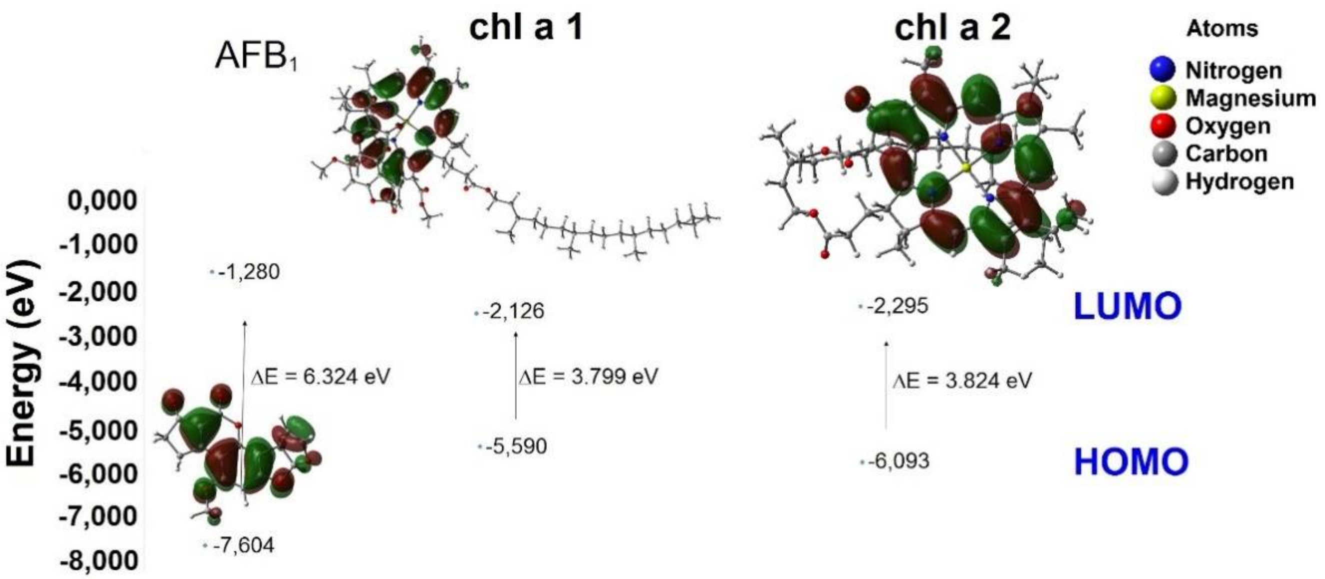

Figure S3. HOMO (eV), LUMO (eV) and the inter-frontier molecular orbital energy gaps of the target molecules.

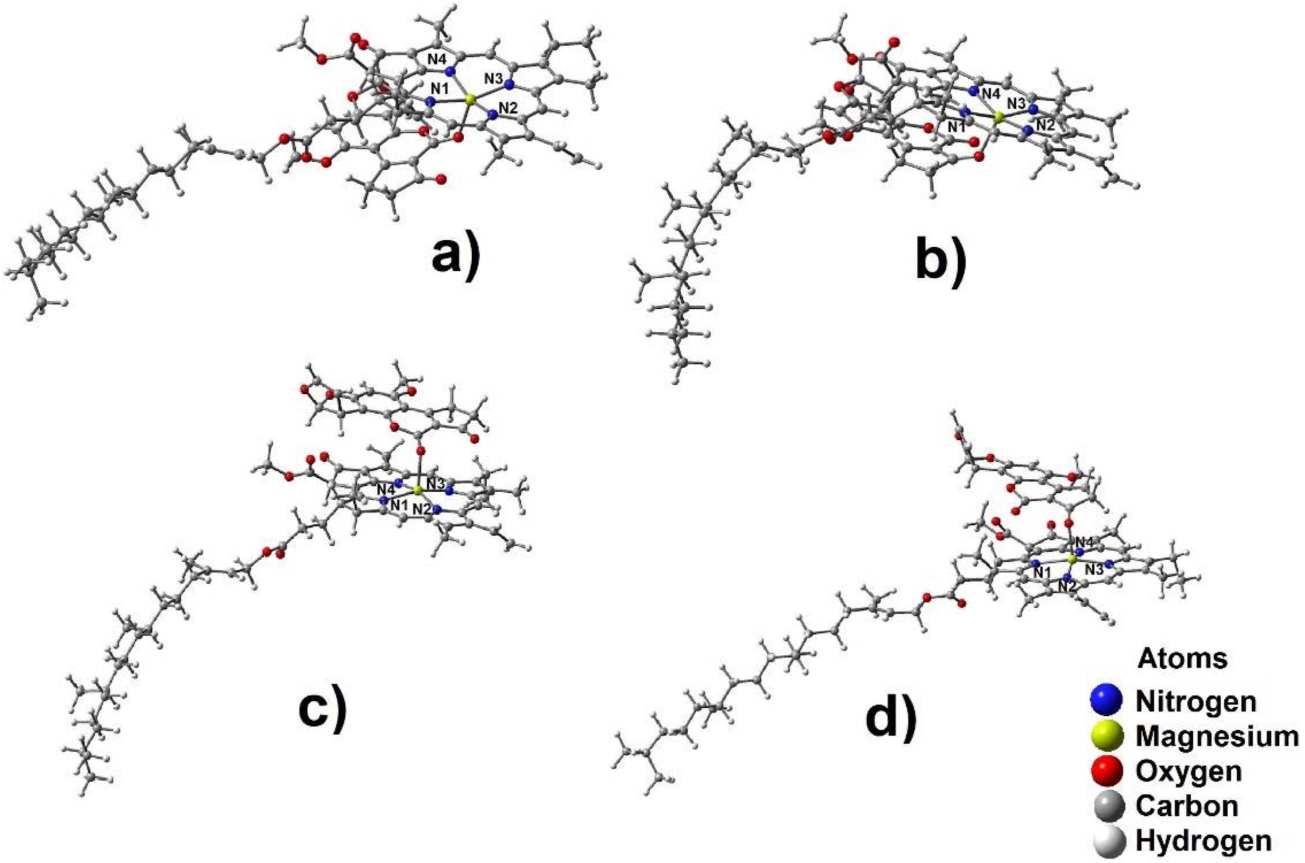

Figure S4. Optimized geometry: a) **chl a 1**-α-D-AFB_1, b) **chl a 1**-α-E-AFB_1, c) **chl a 1**-β-D-AFB_1, and d) **chl a 1**-β-E-AFB_1.

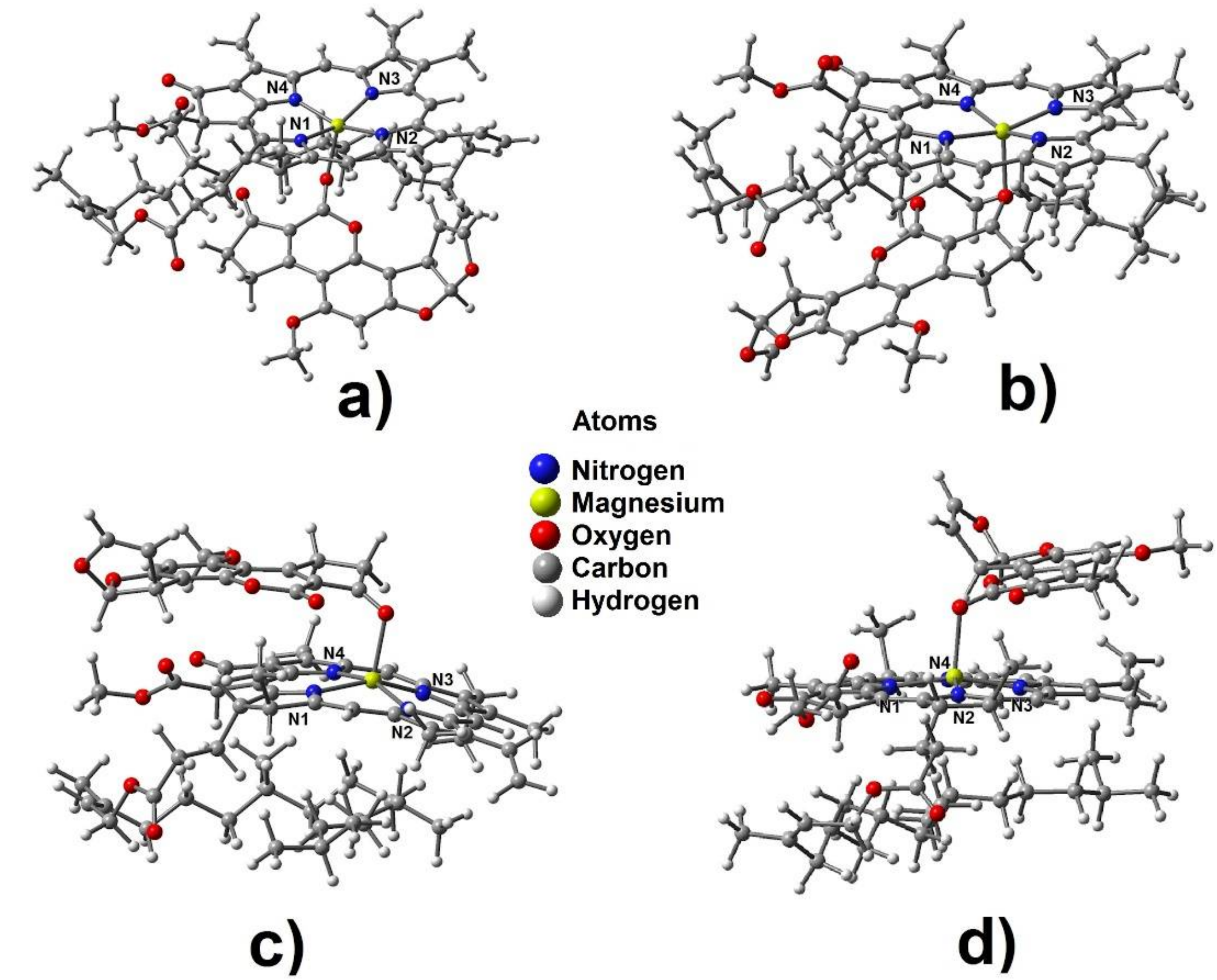

Figure S5. Optimized geometry: a) **chl a 2**-α-D-AFB_1, b) **chl a 2**-α-E-AFB_1, c) **chl a 2**-β-E-AFB_1, and d) **chl a 2**-β-D-AFB_1.

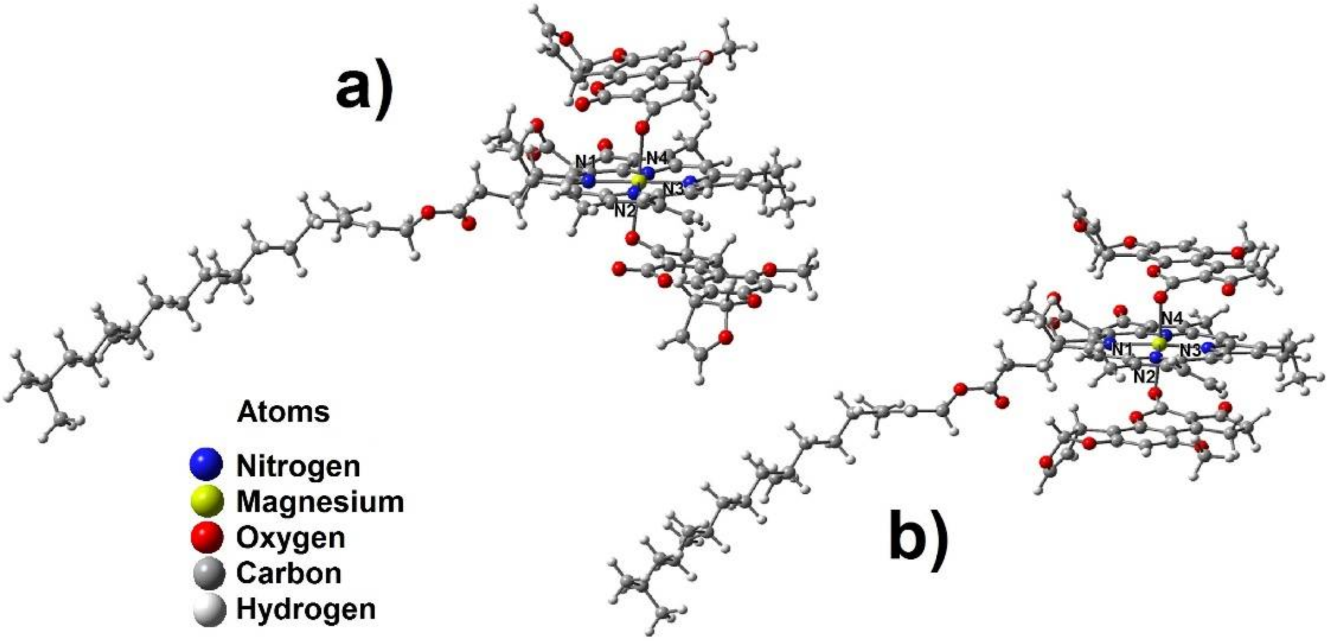

Figure S6. Optimized geometry of a) **chl a 1**-E-2AFB_1, and b) **chl a 1**-D-2AFB_1.

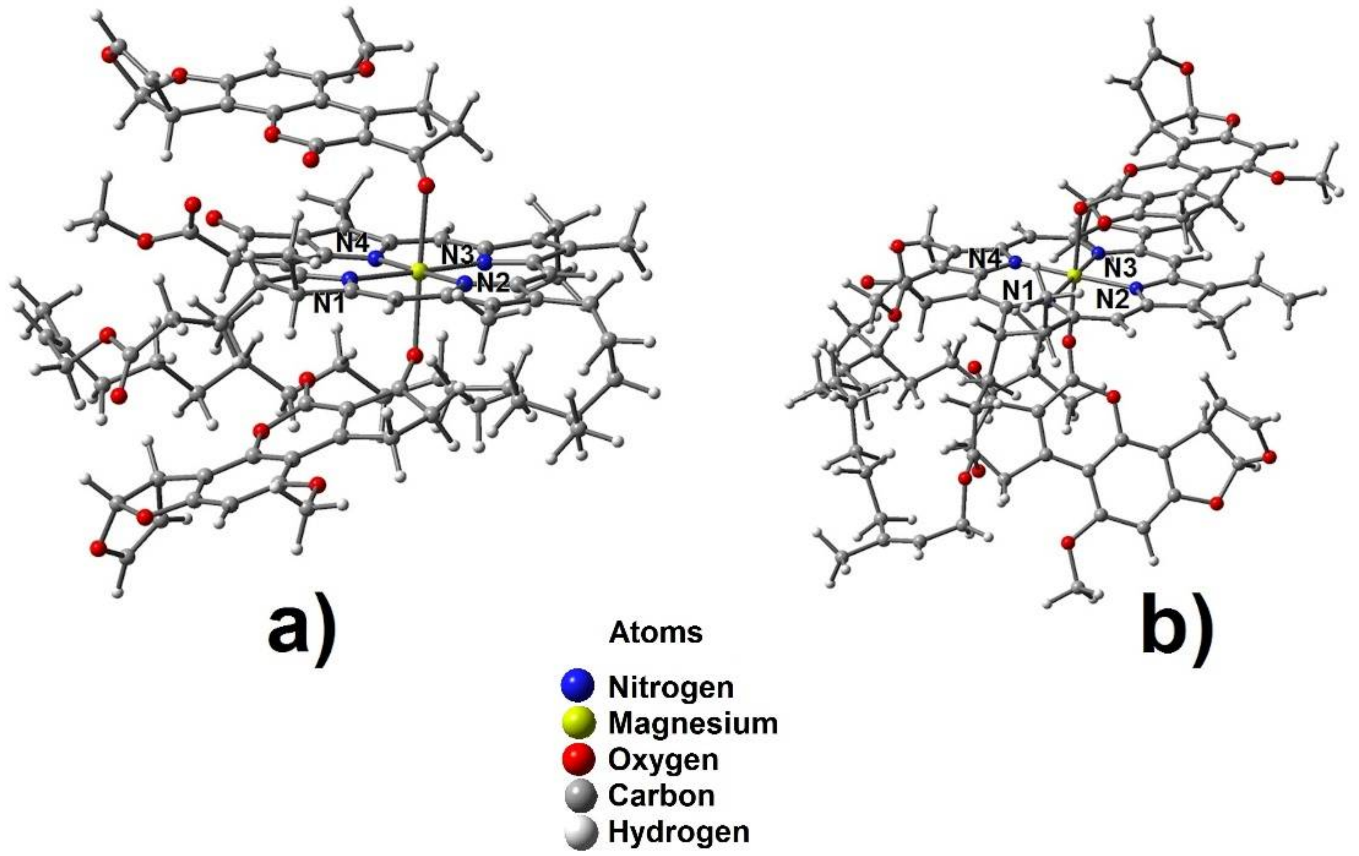

Figure S7. Optimized geometry of a) **chl a 2**-E-2AFB$_1$, and b) **chl a 2**-D-2AFB$_1$.

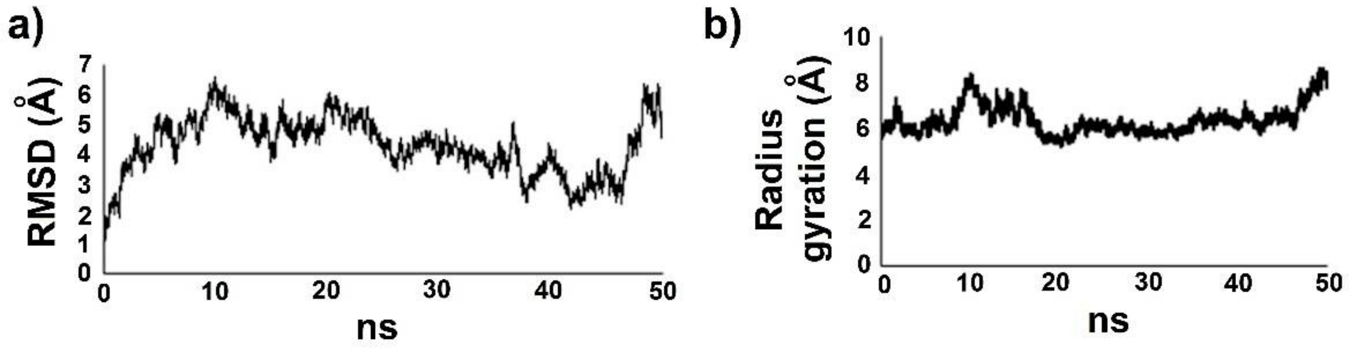

Figure S8. a) RMSD, and b) radius of gyration of **chl a 2**, obtained by MD simulation.

Table S1. Natural atomic charges (e^-) for **chl a 1**, **chl a 2**, AFB_1 and their complexes.

	Mg^{2+}	N1	N2	N3	N4	O*	C*
**chl a 2**	1.762	-0.796	-0.745	-0.770	-0.726		
**chl a 1**	1.758	-0.792	-0.744	-0.769	-0.725		
**chl a 1**-α-D-AFB_1	1.759	-0.763	-0.708	-0.730	-0.693	-0.697	0.873
**chl a 1**-α-E-AFB_1	1.760	-0.698	-0.760	-0.706	-0.734	-0.695	0.646
**chl a 1**-β-E-AFB_1	1.758	-0.767	-0.716	-0.744	-0.691	-0.695	0.641
**chl a 1**-β-D-AFB_1	1.757	-0.763	-0.712	-0.745	-0.694	-0.699	0.868
**chl a 2**-α-D-AFB_1	1.760	-0.763	-0.723	-0.737	-0.690	-0.686	0.877
**chl a 2**-α-E-AFB_1	1.765	-0.746	-0.721	-0.742	-0.695	-0.687	0.648
**chl a 2**-β-E-AFB_1	1.766	-0.779	-0.718	-0.755	-0.704	-0.697	0.643
**chl a 2**-β-D-AFB_1	1.762	-0.768	-0.715	-0.747	-0.691	-0.691	0.870
**chl a 1**-D-2AFB_1	1.753	-0.763	-0.713	-0.727	-0.672	-0.664	0.869
						-0.612	0.853
**chl a 1**-E-2AFB_1	1.753	-0.751	-0.723	-0.737	-0.662	-0.650	0.638
						-0.622	0.624
**chl a 2**-D-2AFB_1	1.749	-0.737	-0.693	-0.711	-0.669	-0.631	0.866
						-0.640	0.865
**chl a 2**-E-2AFB_1	1.756	-0.737	-0.703	-0.726	-0.673	-0.643	0.638
						-0.644	0.642
				E		D	
				O	C	O	C
AFB_1				-0.534	0.588	-0.533	0.825

E= specific oxygen atom of carbonyl group on ring E, D = specific oxygen atom of lactone group on ring D, O* and C*= atoms of AFB_1 when have coordinated to the Mg^{2+} ion.

Table S2. Interaction energy, in kcal/mol, of complexes **chl a 1**-AFB_1, and **chl a 2**-AFB_1 in the gas phase.

**Molecule**	**Interaction energy**	**Energy differences between chlorophylls**	**Energy difference considering carbonylic interaction**
		kcal/mol	
**chl a 1**-α-D-AFB_1	-32.1	4.3	3.6
**chl a 1**-α-E-AFB_1	-36.4	0.0	0.0
**chl a 1**-β-E-AFB_1	-35.5	0.9	0.9
**chl a 1**-β-D-AFB_1	-35.7	0.7	0.0
**chl a 2**-α-D-AFB_1	-33.3	5.9	0.0
**chl a 2**-α-E-AFB_1	-36.5	2.7	2.7
**chl a 2**-β-E-AFB_1	-39.2	0.0	0.0
**chl a 2**-β-D-AFB_1	-32.8	6.4	0.4
**chl a 1**-AFB_1a	-17.0		
**chl a 1**-AFB_1b	-15.6		
**chl a 2**-AFB_1c	-29.6		
**chl a 2**-AFB_1d	-20.4		
**chl a 1**-E-2AFB_1	-60.0	2.2	20.1
**chl a 1**-D-2AFB_1	-62.3	0.0	11.2
**chl a 2**-E-2AFB_1	-64.8	0.0	0.0
**chl a 2**-D-2AFB_1	-58.0	6.7	0.0

a and b = three hydrogen bond interactions, c = one hydrogen bond interaction, d = two hydrogen bond interactions.

Table S3. Interaction energy, in kcal/mol, of complexes **chl a 1**-2AFB_1, and **chl a 2**-2AFB_1 in water as solvent.

Chemical system	Interaction energy	Energy differences between chlorophylls<br><br>kcal/mol	Energy difference considering carbonylic interaction<br><br>kcal/mol
**chl a 1**-α-D-AFB_1	-22.6	3.9	3.8
**chl a 1**-α-E-AFB_1	-26.5	0.0	0.0
**chl a 1**-β-E-AFB_1	-23.6	2.9	2.9
**chl a 1**-β-D-AFB_1	-26.4	0.1	0.0
**chl a 2**-α-D-AFB_1	-23.4	4.5	3.3
**chl a 2**-α-E-AFB_1	-27.6	0.3	0.3
**chl a 2**-β-E-AFB_1	-27.9	0.0	0.0
**chl a 2**-β-D-AFB_1	-26.6	1.3	0.0
**chl a 1**-AFB_1a	-10.3		
**chl a 1**-AFB_1b	-11.2		
**chl a 2**-AFB_1c	-23.0		
**chl a 2**-AFB_1d	-14.4		
**chl a 1**-E-2AFB_1	-45.9	3.1	13.1
**chl a 1**-D-2AFB_1	-49.0	0.0	8.7
**chl a 2**-E-2AFB_1	-47.6	0.0	0.0
**chl a 2**-D-2AFB_1	-46.3	1.3	0.0

a and b = three hydrogen bond interactions, c = one hydrogen bond interaction, d = two hydrogen bond interactions.

Table S4. Cartesian coordinate

AFB_1

0 1			
C	3.97611200	-1.04111100	0.09524100
O	4.66777900	-2.02098700	0.08248700
C	4.47256400	0.39949900	0.25212600
H	5.17443000	0.61354400	-0.55520400
H	5.02225400	0.47604600	1.19130600
C	3.22487300	1.29017000	0.21338200
H	3.23447600	2.01391400	-0.60315100
H	3.07767600	1.86641100	1.12835500
C	2.07363000	0.32835700	0.02858700
C	2.50221600	-0.96072200	-0.03402800
C	1.60571700	-2.09327600	-0.20051100
O	1.86985100	-3.24957000	-0.25583500
O	0.25228000	-1.73577100	-0.30526000
C	0.67733000	0.63841400	-0.07842100
C	-0.18872800	-0.46145400	-0.25480300
C	-1.25721300	2.14418100	-0.13468800
C	-1.54953100	-0.27026500	-0.38316400
H	-1.71504200	3.12013600	-0.09099000
C	-2.05129200	1.01495000	-0.31129700
C	0.11250400	1.94021400	-0.01976300
O	0.99362100	2.94701000	0.15263600
C	0.49297100	4.27122500	0.21944200
H	-0.01816500	4.54245300	-0.70826600
H	1.36150800	4.91008700	0.35814700
H	-0.18832300	4.38899400	1.06649900
O	-3.39296700	1.07027500	-0.44604000
O	-4.80161600	-0.57873700	0.38475400
C	-3.03731200	-1.97129500	0.75221800
H	-2.40863500	-2.71018300	1.22159900
C	-4.20450000	-1.52249000	1.18749400
H	-4.77845600	-1.78905500	2.06201400
C	-2.69127000	-1.23606600	-0.52301500
H	-2.58178100	-1.90080300	-1.38165300
C	-3.89967800	-0.25926300	-0.64292000
H	-4.44188700	-0.25572600	-1.58587500

chl a 2

0 1							
Mg	-1.74528200	-0.15458700	-1.10972300	H	2.12533400	-5.23729100	-1.52614700
N	-3.08417400	-1.65161200	-0.97566800	H	0.99765900	-4.45282500	-2.64258300
C	-4.45654900	-1.52295200	-1.02086700	C	3.15220800	-2.36470700	0.13230300
C	-5.05786300	-2.85027100	-0.88980800	H	2.68758500	-2.20283900	1.11196700
C	-4.03165600	-3.72964200	-0.69769600	H	3.78170400	-1.49761400	-0.06882300
C	-2.80105500	-2.95694400	-0.77370300	C	4.06838300	-3.59282400	0.18075200
C	-6.48461100	-3.17542400	-0.95313400	H	3.54429500	-4.48384000	0.52829100
H	-6.83396100	-3.94041600	-0.26300600	H	4.46668500	-3.77818600	-0.82212100
C	-7.35461500	-2.62735000	-1.80074200	C	5.22463600	-3.34047700	1.11801700
H	-8.39701900	-2.92165600	-1.79818600	O	5.43780200	-3.91968300	2.14672600
H	-7.04371800	-1.89267700	-2.53511700	O	5.98408000	-2.32852000	0.66379600
C	-4.09854200	-5.20664400	-0.48434900	C	7.06058300	-1.90546900	1.53416300
H	-3.62961900	-5.48519300	0.46385600	H	7.72771700	-2.75590000	1.68870800
H	-3.57636900	-5.74148900	-1.28252400	H	6.63086700	-1.63737600	2.50026400
H	-5.13086800	-5.55612500	-0.46985300	C	7.74745900	-0.76469400	0.85860300
C	-5.13612200	-0.32542000	-1.04273700	C	7.52467600	0.52880800	1.10244400
H	-6.21658400	-0.38066700	-0.97696400	H	8.43728600	-1.03515700	0.06324400
N	-3.25886600	1.23974400	-1.22711000	C	8.18194200	1.59351900	0.26784300
C	-4.57763000	0.97493800	-1.10088000	H	7.42016900	2.16138500	-0.27787900
C	-3.13268500	2.60776700	-1.29470300	H	8.87749800	1.16772300	-0.45670400
C	-4.43573800	3.22236200	-1.17464600	H	8.72696200	2.30231100	0.89767800
C	-5.34005900	2.19946700	-1.04577700	C	6.55098200	1.03760700	2.14106000
C	-6.82388200	2.27612400	-0.86439900	H	6.51619300	0.37949000	3.01295300
H	-7.13381000	1.77141600	0.05502200	H	6.89182100	2.01427200	2.50020300
H	-7.35240400	1.79906900	-1.69477600	C	5.14150200	1.18439700	1.55409300
H	-7.16066600	3.31142400	-0.80498200	H	4.82132300	0.19379500	1.20943500
C	-4.68631900	4.69785000	-1.11731800	H	5.19631800	1.81565600	0.65991900
H	-5.72142100	4.90625600	-1.39810300	C	4.11241600	1.74089000	2.54376900
H	-4.06282000	5.20952300	-1.85628300	H	3.89486000	0.97239400	3.29404100
C	-4.40502100	5.26504400	0.28065700	H	4.54116900	2.59332500	3.08640700
H	-4.56874600	6.34414200	0.31160600	C	2.81219800	2.18790600	1.85716500
H	-3.37276400	5.06102900	0.57702700	H	2.54460700	1.42056200	1.11859300
H	-5.05683900	4.79397100	1.01970700	C	1.60836800	2.28773800	2.80266100
C	-1.92818800	3.29299400	-1.47066500	H	1.68120600	1.53283900	3.59346500
H	-1.98606200	4.37288900	-1.56932600	H	1.61676600	3.26399500	3.30459900
C	-0.65904600	2.72024800	-1.53535300	C	0.28074300	2.09232400	2.06089800
N	-0.45931500	1.35904700	-1.34977800	H	0.18315100	1.03006200	1.80461900
C	-1.51552100	-3.50395900	-0.65682600	H	0.29791500	2.63039300	1.10483300
H	-1.46629700	-4.56980500	-0.46862100	C	-0.91848800	2.56987200	2.87572400
C	-0.29809200	-2.84551200	-0.77250800	H	-0.82029400	2.21748600	3.91204200
N	-0.12118200	-1.52865300	-0.98851500	H	-0.88137900	3.66467500	2.92425000
C	1.22357200	-1.23803800	-1.07905500	C	-2.29478900	2.13425200	2.35543400
C	0.84848200	1.16621200	-1.45065200	H	-2.36742800	2.39371200	1.28997200
C	1.68421600	0.02973700	-1.31192100	C	-2.46986700	0.60872500	2.49759400
C	0.60002000	3.38035500	-1.77633700	H	-1.89977200	0.09289300	1.71083600
C	1.54421000	2.36830000	-1.72630300	H	-2.01042100	0.29155700	3.44413700
C	0.82060300	4.83874900	-2.01229200	C	-3.91197400	0.10344000	2.47800000
H	0.25679000	5.18665000	-2.88149400	H	-4.42409300	0.47842800	1.58401500
H	1.87878600	5.03111000	-2.18712100	H	-4.44396900	0.52020400	3.34089700
H	0.49932300	5.42890100	-1.14904400	C	-4.00314100	-1.42263200	2.53650000
C	2.97261000	2.06607200	-1.83412900	H	-3.78413400	-1.84410500	1.54864400
O	3.90143000	2.76643700	-2.13928700	H	-3.22689000	-1.80420600	3.21180000
C	3.10640100	0.56038400	-1.38711400	C	-5.35890200	-1.95261200	3.02198700
H	3.53555600	0.64680800	-0.38694400	H	-5.46808600	-1.66099300	4.07483600
C	4.12158000	-0.21789200	-2.18328700	C	3.02007500	3.52539000	1.13770500
O	5.35114500	0.09034700	-1.75840500	H	3.80287100	3.48043300	0.37857500
C	6.42484500	-0.61887300	-2.37709900	H	3.30505700	4.28711000	1.87065800
H	6.45642100	-1.63728300	-1.98571700	H	2.10497700	3.85968900	0.64343700
H	7.32930800	-0.08425500	-2.09826900	C	-3.38313500	2.90797600	3.10452800
H	6.29774600	-0.63529900	-3.45882000	H	-3.19504600	3.98278700	3.04637000
O	3.88604800	-1.02546500	-3.04160300	H	-3.39863800	2.62833200	4.16392400
C	1.02523700	-3.58727500	-0.65911800	H	-4.37556200	2.72058000	2.68932400
H	1.12300900	-3.94385300	0.37410700	C	-5.38861400	-3.47886300	2.93934000
C	2.06736400	-2.48702600	-0.93738900	H	-5.30483300	-3.79592700	1.89405500
H	2.55636500	-2.66706500	-1.90162700	H	-6.32002500	-3.88322700	3.34414500
C	1.13643200	-4.77971100	-1.60949800	H	-4.55606400	-3.92368900	3.49111100
H	0.39109600	-5.54638200	-1.39079600	C	-6.53223900	-1.35492000	2.24462900
				H	-6.44327100	-1.58767300	1.18031000
				H	-6.57516100	-0.26801700	2.34797100
				H	-7.48164700	-1.76369200	2.60094600

chl a 1-α-E-AFB_1

0 1							
Mg	-5.04151200	2.62023600	0.24818000	H	5.46154600	-4.05418300	1.74152200
N	-4.55355000	4.59721000	0.30025000	H	7.01901200	-4.04542500	2.57864900
C	-5.38757900	5.62853700	-0.06678300	C	7.96261100	-1.64028700	1.87880100
C	-4.61690200	6.87719900	-0.08268400	H	7.91616500	-0.54855300	1.83306700
C	-3.35579000	6.56230200	0.32406000	H	8.21533100	-1.90779500	2.91304700
C	-3.33745700	5.11927200	0.54529200	C	9.08215800	-2.13734200	0.95534600
C	-5.10438100	8.20610900	-0.46202000	H	8.81804300	-1.90032700	-0.08263800
H	-4.75319100	9.03663200	0.14574500	H	9.15319700	-3.22682700	1.02286500
C	-5.90202200	8.46382900	-1.49768800	C	10.42934800	-1.50455200	1.30075100
H	-6.21359700	9.47648700	-1.72307600	H	10.29413700	-0.42028400	1.39277400
H	-6.24334900	7.67694900	-2.16088100	H	10.75472300	-1.85868500	2.28812200
C	-2.17689000	7.46533400	0.49010400	C	11.54747100	-1.76853300	0.28476900
H	-1.35837600	7.16499900	-0.17009100	H	11.21615600	-1.37289600	-0.68554100
H	-1.79966900	7.43468800	1.51615800	C	12.80947400	-1.00226500	0.69890200
H	-2.43436200	8.49724600	0.25088300	H	12.53565900	0.04204300	0.89177100
C	-6.73985700	5.51419100	-0.29551000	H	13.17134300	-1.40533800	1.65454000
H	-7.27187800	6.43012800	-0.52177400	C	13.94821400	-1.02513300	-0.32045000
N	-7.05078500	3.11842900	0.09254400	H	13.57169700	-0.68048300	-1.29240000
C	-7.52610700	4.33463400	-0.21926200	H	14.29573200	-2.05161900	-0.46355900
C	-8.12147000	2.25635000	0.08753500	C	15.11913400	-0.14235700	0.11183600
C	-9.32719000	2.98062300	-0.26769400	H	14.73214800	0.85004500	0.37260000
C	-8.95616700	4.28272300	-0.45665600	H	15.55808300	-0.55140100	1.03182900
C	-9.80305400	5.45194600	-0.85120400	C	16.23007700	0.03019400	-0.93072500
H	-9.46211900	5.88447400	-1.79619700	H	15.77811900	0.45756200	-1.83668400
H	-9.76760100	6.24239500	-0.09627400	C	17.26860000	1.03043100	-0.40855500
H	-10.84510600	5.15716000	-0.97882900	H	16.74977900	1.95493000	-0.12663800
C	-10.67380700	2.35621400	-0.46629700	H	17.70910800	0.63301300	0.51562200
H	-11.45287300	3.11305700	-0.34665600	C	18.38787100	1.38126800	-1.38894800
H	-10.85474900	1.60838500	0.31133500	H	17.94534700	1.64186500	-2.35721800
C	-10.78951700	1.70391900	-1.85129700	H	19.02823500	0.50902200	-1.56177800
H	-11.76190400	1.22389300	-1.98238000	C	19.25233600	2.53482700	-0.88091400
H	-10.00545200	0.95632300	-1.99255900	H	18.64908500	3.45197500	-0.84536800
H	-10.66705500	2.45662300	-2.63313100	H	19.55007500	2.32331100	0.15347500
C	-8.06147600	0.90346700	0.39707900	C	20.51317600	2.80502800	-1.70791900
H	-8.98969800	0.34383700	0.33498000	H	21.12154200	1.89149300	-1.69903600
C	-6.91759700	0.18398500	0.76427900	C	11.82057500	-3.26489900	0.12352400
N	-5.67273200	0.78492200	0.86350500	H	10.93114900	-3.80343100	-0.20892800
C	-2.20408000	4.40350700	0.96211900	H	12.14064200	-3.69624900	1.07838700
H	-1.30279400	4.98459800	1.12042200	H	12.60535300	-3.45322600	-0.61085100
C	-2.10649700	3.04045500	1.21759600	C	16.87370700	-1.30649100	-1.30218200
N	-3.07939900	2.12895600	1.06892000	H	16.14231900	-2.00801400	-1.70724700
C	-2.61299500	0.87492500	1.39400300	H	17.33029200	-1.76567700	-0.41848100
C	-4.82453900	-0.18631200	1.15706200	H	17.65278500	-1.17912500	-2.05583100
C	-3.42010800	-0.22924600	1.37263100	C	21.33049000	3.93046500	-1.07373300
C	-6.81668000	-1.22331500	1.01438200	H	20.75808100	4.86360700	-1.07712100
C	-5.46456700	-1.44447200	1.26163100	H	22.25886100	4.10596100	-1.62247500
C	-7.92176400	-2.22652300	0.96862600	H	21.58729600	3.69893800	-0.03695800
H	-8.69766700	-2.00329800	1.70543700	C	20.18106200	3.14352700	-3.16189500
H	-7.52390600	-3.22050600	1.17958300	H	19.51309400	4.01046300	-3.20617000
H	-8.38891000	-2.23272700	-0.02134800	H	19.69078000	2.31346600	-3.67400800
C	-4.46840200	-2.45813100	1.57123000	H	21.08686000	3.39134000	-3.72042900
O	-4.57059300	-3.64678700	1.74838100	C	-3.94776700	0.91150300	-1.92018900
C	-3.07759400	-1.69164300	1.60297800	O	-4.49740700	1.97605600	-1.68154000
H	-2.49516600	-2.12864400	0.78620500	C	-2.45018800	0.73739100	-2.07751400
C	-2.34770400	-2.00434800	2.88432900	H	-1.93653700	1.37447600	-1.35551500
O	-1.66853300	-3.15893300	2.77203600	H	-2.17632300	1.07868300	-3.07976900
C	-1.02774800	-3.60236900	3.96820500	C	-2.20501000	-0.76206900	-1.88157100
H	-0.52882300	-4.53295000	3.71114900	H	-1.67968800	-0.97315200	-0.95173200
H	-1.76787400	-3.76727100	4.75136200	H	-1.61803900	-1.23069400	-2.67375300
H	-0.30672100	-2.85968400	4.31183800	C	-3.58635900	-1.36549600	-1.84311100
O	-2.35288500	-1.34040400	3.88432000	C	-4.55499900	-0.40919400	-1.99364300
C	-0.80797800	2.41590500	1.71439300	C	-5.97250800	-0.71988800	-2.09754400
H	-0.04082300	2.55638300	0.94199900	O	-6.87498600	0.02276100	-2.32181900
C	-1.16543600	0.92103000	1.84976200	O	-6.26488300	-2.08283000	-1.97696000
H	-1.15142400	0.62994400	2.90721400	C	-3.95045800	-2.72211400	-1.62192500
C	-0.31643700	3.03799300	3.02259200	C	-5.33533000	-3.01695300	-1.66813600
H	-0.08731400	4.09827000	2.90326000	C	-3.48297300	-5.03512300	-0.96395300
H	0.58648900	2.53098400	3.37276800	C	-5.78177300	-4.28893800	-1.39158700
H	-1.08381700	2.94036500	3.79407200	H	-2.82269200	-5.82691600	-0.64675500
C	-0.22896800	-0.01327800	1.08303300	C	-4.85198100	-5.26138300	-1.05231500
H	-0.17461100	0.29856500	0.03424700	C	-3.04184300	-3.75430500	-1.25269300
H	-0.61007100	-1.03747800	1.08407000	O	-1.75456600	-3.37759800	-1.16027600
C	1.17521200	-0.03952700	1.67038800	C	-0.84521500	-4.23271700	-0.46996200
H	1.67412700	0.93127200	1.60771900	H	-1.23805300	-4.46328500	0.52447400
H	1.14359100	-0.29971400	2.73504800	H	0.07830200	-3.66416700	-0.38302000
C	2.04843400	-1.06280800	0.98422400	H	-0.67334500	-5.15066400	-1.03823500
O	1.65254400	-1.93016800	0.24824700	O	-5.40917200	-6.45756700	-0.79507100
O	3.33534000	-0.89961200	1.30176900	O	-7.30299800	-7.26611400	-1.86175300
C	4.25819500	-1.82377300	0.68963600	C	-7.63021500	-5.23601100	-2.83795300
H	4.13357300	-1.75293000	-0.39531200	H	-7.87965900	-4.49738900	-3.58219600
H	3.99177400	-2.83902600	0.98466100	C	-7.66533200	-6.55240600	-2.97939000
C	5.62970600	-1.41755600	1.11507100	H	-7.94978600	-7.16140000	-3.82404700
C	6.61030900	-2.21622300	1.53886200	C	-7.14385600	-4.92046000	-1.44090000
H	5.82159300	-0.35032000	1.03686400	H	-7.88459300	-4.38645800	-0.84571400
C	6.49358500	-3.71184600	1.67969400	C	-6.83734500	-6.34937200	-0.90605900
H	6.96154800	-4.21739300	0.82976700	H	-7.25362900	-6.61667100	0.06254200

chl a 2-β-E-AFB_1

0 1							
Mg	2.35799400	-0.91822900	-0.94294600	H	-6.50999900	1.47613200	4.57445000
N	3.69081000	0.40553000	-1.70689000	H	-5.55632900	1.55005100	6.06630900
C	5.05630800	0.33218000	-1.61401900	C	-3.18853700	2.49872600	4.88717900
C	5.65223600	1.35760700	-2.48003600	H	-2.77241300	3.50933200	4.89376900
C	4.62172300	1.99807400	-3.09885900	H	-3.17936700	2.15249700	5.92599900
C	3.39165000	1.40167300	-2.56600700	C	-2.28208700	1.58467300	4.05138100
C	7.08951100	1.57966000	-2.63145600	H	-2.30576900	1.94599000	3.01615900
H	7.72363600	0.69720800	-2.59923100	H	-2.71047100	0.57585400	4.03573600
C	7.65304400	2.77585400	-2.79589300	C	-0.83712100	1.55755800	4.55518300
H	8.72443100	2.88075100	-2.91562400	H	-0.38046100	2.53378500	4.35474000
H	7.06020300	3.68398000	-2.79238300	H	-0.83088300	1.43189700	5.64596900
C	4.66913200	3.04898900	-4.15994100	C	0.02350900	0.44901900	3.93092200
H	4.52075000	4.05223100	-3.74985300	H	-0.15158000	0.44674800	2.84366600
H	3.88829100	2.87688100	-4.90385800	C	1.52173500	0.71609000	4.12197200
H	5.63606000	3.03220900	-4.66541500	H	1.72157500	1.76073100	3.87503400
C	5.75675900	-0.54380600	-0.81755900	H	1.78085400	0.59867200	5.18288100
H	6.83650500	-0.44733500	-0.81227000	C	2.41748000	-0.19745400	3.27077900
N	3.90435200	-1.82239500	0.15070100	H	1.96302500	-0.33428400	2.28078600
C	5.21200100	-1.54602800	0.02767900	H	2.44270000	-1.19586300	3.71929500
C	3.79537000	-2.82755700	1.08868900	C	3.85764300	0.30746800	3.09138500
C	5.11986300	-3.20103500	1.55780900	H	4.17625300	0.83624000	4.00096700
C	6.00394400	-2.39096400	0.90541900	H	4.52951100	-0.55308400	2.98178600
C	7.49155600	-2.32186800	1.04879300	C	4.08942200	1.21727100	1.87291000
H	7.80948000	-1.33623400	1.40101400	H	3.93056700	0.60678100	0.97743200
H	7.99176400	-2.50663400	0.09406100	C	3.09823900	2.37975000	1.78535500
H	7.85254100	-3.06137100	1.76456600	H	2.08492900	1.96960400	1.67549000
C	5.40201800	-4.19723300	2.63982200	H	3.10544300	2.95087700	2.72349400
H	6.40419200	-4.61164600	2.50512900	C	3.35171600	3.34654800	0.62707600
H	4.71103400	-5.04116100	2.55866300	H	3.64518700	2.78155500	-0.26567200
C	5.28753600	-3.56376600	4.03322000	H	4.19371500	4.00483000	0.87212900
H	5.46665300	-4.29928300	4.82028600	C	2.10716600	4.18494400	0.32600600
H	4.29314800	-3.13394800	4.17833800	H	1.41559300	3.56969500	-0.26584800
H	6.01357500	-2.75506200	4.14318500	H	1.58947200	4.41452000	1.26648600
C	2.60561000	-3.34755900	1.57179300	C	2.35911300	5.49933500	-0.41585400
H	2.68288400	-4.13636500	2.31449200	H	2.97258500	6.13521900	0.23551800
C	1.31008100	-2.88409400	1.27819100	C	-0.36120400	-0.91960200	4.49985800
N	1.08962400	-1.84249400	0.38536100	H	-1.39791100	-1.19138200	4.28258200
C	2.10887500	1.88055200	-2.82962400	H	-0.23588500	-0.90970700	5.58797100
H	2.03427200	2.71007800	-3.52253600	H	0.27403200	-1.70664300	4.09269400
C	0.92146700	1.51036800	-2.19449500	C	5.54181600	1.69449400	1.85157100
N	0.75229300	0.48721500	-1.34874900	H	6.22205000	0.84352200	1.95627800
C	-0.52120700	0.53208400	-0.80998800	H	5.73510000	2.38767900	2.67817000
C	-0.18369700	-1.49718400	0.54652600	H	5.78920800	2.19963700	0.91455400
C	-0.97578500	-0.40723100	0.07519700	C	1.03601700	6.21634200	-0.68494900
C	0.10015700	-3.23759000	1.95197400	H	0.41752300	5.62350500	-1.36835100
C	-0.84574600	-2.33268000	1.47254900	H	1.19575500	7.19328100	-1.14786700
C	-0.09083700	-4.29937800	2.98714000	H	0.46083200	6.36279000	0.23270500
H	-0.05493100	-5.29937800	2.54352200	C	3.12438300	5.27151100	-1.71783800
H	-1.06094600	-4.17600700	3.46986100	H	2.53930100	4.63006400	-2.38480800
H	0.68606800	-4.25176800	3.75505800	H	4.08232500	4.77566200	-1.53794900
C	-2.20468600	-1.85139900	1.66009400	H	3.31599200	6.21596700	-2.23438600
O	-3.14078100	-2.34786700	2.23970200	C	1.18186100	-3.05783900	-2.36538600
C	-2.20692700	-0.42618600	0.98567900	O	2.19538800	-2.37920500	-2.47073000
H	-1.97383200	0.20084200	1.85716300	C	1.11124300	-4.43644700	-1.74553500
C	-3.55502000	0.08240600	0.55340500	H	1.84742700	-4.52250600	-0.94583300
O	-4.36904800	0.16061400	1.60279600	H	1.35764400	-5.16673300	-2.52234300
C	-5.68331800	0.64871700	1.32286100	C	-0.34630500	-4.56440400	-1.27778900
H	-5.62273100	1.66660600	0.93433700	H	-0.42940900	-4.46798300	-0.19402800
H	-6.20498900	0.63745600	2.27590500	H	-0.82801900	-5.50501500	-1.54890600
H	-6.17701800	-0.00713200	0.60326000	C	-1.05452600	-3.40787200	-1.93646500
O	-3.87606600	0.44048800	-0.55420600	C	-0.17713700	-2.63714200	-2.65352100
C	-0.33966400	2.34327400	-2.36313000	C	-0.57207900	-1.48674600	-3.44095300
H	-0.09700500	3.38362600	-2.11419600	O	0.11594300	-0.78492800	-4.11723700
C	-1.28458200	1.74052200	-1.30841200	O	-1.94089500	-1.23302500	-3.42411500
H	-2.21624100	1.40223300	-1.76786800	C	-2.42000400	-3.02296800	-1.84592600
C	-0.90788200	2.29871700	-3.78366900	C	-2.80921300	-1.90899100	-2.63059200
H	-0.18734500	2.67667600	-4.51201900	C	-4.66422700	-3.08991800	-0.86385900
H	-1.80742500	2.91850900	-3.83809500	C	-4.09887900	-1.44487600	-2.59067200
H	-1.16104900	1.27516300	-4.06295200	H	-5.38814800	-3.45975400	-0.15420800
C	-1.63232000	2.69292900	-0.16191200	C	-4.98471400	-2.02208700	-1.69530200
H	-0.72193200	3.17351000	0.21289000	C	-3.37332300	-3.59749100	-0.95959500
H	-2.05649900	2.13114800	0.67180500	O	-2.92203400	-4.61701100	-0.21193900
C	-2.65585700	3.75576700	-0.57687500	C	-3.78383200	-5.15282800	0.78594700
H	-2.23758700	4.46360300	-1.29401600	H	-4.05487500	-4.37761600	1.50490500
H	-3.52402000	3.25359200	-1.01345700	H	-3.20658400	-5.92957400	1.28152000
C	-3.09207100	4.51871500	0.64646700	H	-4.67389900	-5.59311400	0.32784900
O	-2.75325400	5.63000200	0.94731200	O	-6.16590800	-1.37713500	-1.63714300
O	-3.88284000	3.75160800	1.42244900	O	-7.13762200	-0.21752900	-3.39762200
C	-4.25239800	4.32729900	2.69690500	C	-5.25788200	-0.52035000	-4.64562200
H	-4.81587000	5.24288100	2.50504200	H	-4.62922200	-0.71086900	-5.50015700
H	-3.33902400	4.60267500	3.22520900	C	-6.58233100	-0.49380700	-4.62526700
C	-5.06605000	3.30888700	3.42554200	H	-7.30218300	-0.63386400	-5.41738400
C	-4.61242100	2.49327800	4.38045200	C	-4.75602300	-0.26667600	-3.24247600
H	-6.09884400	3.21047800	3.10054100	H	-4.15397300	0.63881100	-3.14663600
C	-5.49804200	1.43593100	4.98000500	C	-6.09552100	-0.19396300	-2.45891200
H	-5.08129300	0.44230200	4.78099100	H	-6.22705800	0.65926700	-1.79834300

chl a 1-D-2AFB_1

0 1							
Mg	-4.16600000	1.94300000	-0.51100000	H	15.97800000	-0.61800000	0.32700000
N	-3.87000000	3.91900000	-0.17900000	C	16.48800000	1.07700000	1.56200000
C	-4.80500000	4.90900000	-0.35500000	H	15.99000000	1.98800000	1.91600000
C	-4.27700000	6.16300000	0.20000000	H	16.68600000	0.46900000	2.45500000
C	-3.05500000	5.87300000	0.72500000	C	17.82600000	1.47000000	0.92300000
C	-2.81700000	4.45400000	0.46600000	H	17.60600000	2.03200000	0.00500000
C	-4.92800000	7.47600000	0.17700000	C	18.59800000	2.40100000	1.86600000
H	-4.83200000	8.07200000	1.08100000	H	17.95700000	3.25700000	2.11200000
C	-5.58600000	7.98800000	-0.86200000	H	18.78400000	1.87500000	2.81200000
H	-6.03700000	8.97100000	-0.80800000	C	19.92200000	2.92600000	1.31300000
H	-5.67100000	7.45000000	-1.79900000	H	19.75500000	3.31500000	0.30200000
C	-2.08800000	6.78900000	1.40000000	H	20.64400000	2.10700000	1.21700000
H	-1.14600000	6.84000000	0.84500000	C	20.52800000	4.01400000	2.19900000
H	-1.85600000	6.44000000	2.41000000	H	19.87400000	4.89700000	2.18800000
H	-2.48700000	7.80100000	1.47100000	H	20.54400000	3.65700000	3.23600000
C	-6.04900000	4.73800000	-0.91600000	C	21.94500000	4.44500000	1.80800000
H	-6.70500000	5.60000000	-0.91400000	H	22.59100000	3.56000000	1.86300000
N	-5.92500000	2.37900000	-1.56700000	C	13.52200000	-1.85500000	-0.22400000
C	-6.56800000	3.55400000	-1.50300000	H	12.76600000	-2.24500000	-0.90800000
C	-6.75000000	1.50700000	-2.24400000	H	13.67400000	-2.59400000	0.57000000
C	-7.97500000	2.19000000	-2.62000000	H	14.45200000	-1.76800000	-0.78700000
C	-7.86500000	3.46800000	-2.15000000	C	18.65600000	0.24200000	0.54700000
C	-8.85800000	4.58300000	-2.21900000	H	18.10900000	-0.42900000	-0.11800000
H	-8.42900000	5.47300000	-2.68800000	H	18.92600000	-0.32100000	1.44700000
H	-9.18800000	4.86200000	-1.21400000	H	19.57900000	0.52300000	0.03600000
H	-9.73700000	4.29600000	-2.79800000	C	22.47300000	5.48500000	2.79600000
C	-9.09000000	1.59000000	-3.42200000	H	21.85700000	6.38900000	2.76200000
H	-10.02600000	2.10900000	-3.19400000	H	23.50000000	5.77300000	2.55800000
H	-9.24200000	0.54500000	-3.12900000	H	22.45600000	5.10600000	3.82000000
C	-8.81700000	1.66300000	-4.93000000	C	22.00200000	4.99100000	0.38100000
H	-9.63400000	1.21900000	-5.50300000	H	21.31100000	5.83400000	0.26900000
H	-7.89200000	1.13700000	-5.17700000	H	21.73200000	4.23400000	-0.35800000
H	-8.69900000	2.70200000	-5.24100000	H	23.00600000	5.34800000	0.14000000
C	-6.46000000	0.17200000	-2.49500000	C	-8.10700000	1.27400000	0.63200000
H	-7.20500000	-0.40300000	-3.03800000	O	-7.96100000	2.43900000	0.88300000
C	-5.29500000	-0.50700000	-2.11100000	C	-9.39300000	0.62300000	0.11800000
N	-4.25400000	0.13200000	-1.44900000	H	-9.74200000	1.17100000	-0.75800000
C	-1.65200000	3.77000000	0.83400000	H	-10.15300000	0.72200000	0.89700000
H	-0.89200000	4.35400000	1.34200000	C	-9.04600000	-0.84400000	-0.16300000
C	-1.33600000	2.43400000	0.59600000	H	-9.02600000	-1.07400000	-1.23300000
N	-2.11100000	1.52400000	-0.00900000	H	-9.73400000	-1.55900000	0.29100000
C	-1.43300000	0.32900000	-0.13600000	C	-7.65000000	-1.02200000	0.38200000
C	-3.30600000	-0.78100000	-1.30000000	C	-7.12800000	0.17200000	0.79500000
C	-1.99500000	-0.76200000	-0.74100000	C	-5.75200000	0.29200000	1.18300000
C	-4.97700000	-1.88600000	-2.33900000	O	-5.10000000	1.31300000	1.29000000
C	-3.69700000	-2.04100000	-1.81600000	O	-5.09600000	-0.88000000	1.41500000
C	-5.83700000	-2.90400000	-3.01800000	C	-6.87300000	-2.21400000	0.45200000
H	-6.87200000	-2.84000000	-2.67000000	C	-5.59400000	-2.08500000	1.03400000
H	-5.45900000	-3.90600000	-2.81400000	C	-6.43700000	-4.60400000	0.12700000
H	-5.84000000	-2.75300000	-4.10100000	C	-4.77400000	-3.17300000	1.20400000
C	-2.64100000	-3.00000000	-1.54200000	H	-6.69800000	-5.58600000	-0.23700000
O	-2.64100000	-4.20600000	-1.59900000	C	-5.20200000	-4.40600000	0.74500000
C	-1.39900000	-2.12300000	-1.08600000	C	-7.26500000	-3.50000000	-0.01100000
H	-0.78700000	-2.05800000	-1.99500000	O	-8.47100000	-3.55000000	-0.62000000
C	-0.55300000	-2.83100000	-0.06000000	C	-8.89200000	-4.79200000	-1.15900000
O	0.15900000	-3.80500000	-0.62900000	H	-8.19300000	-5.13700000	-1.92600000
C	0.93200000	-4.60400000	0.27000000	H	-9.86500000	-4.60600000	-1.60600000
H	1.40900000	-5.35900000	-0.34800000	H	-8.98500000	-5.54700000	-0.37400000
H	0.28300000	-5.06600000	1.01600000	O	-4.29400000	-5.37700000	0.91900000
H	1.67700000	-3.99000000	0.77600000	O	-2.66300000	-5.44900000	2.57300000
O	-0.50600000	-2.58900000	1.12000000	C	-3.37500000	-3.41500000	3.30300000
C	0.00500000	1.85500000	1.02000000	H	-3.63700000	-2.61600000	3.97700000
H	0.80100000	2.41900000	0.52000000	C	-2.96200000	-4.62900000	3.63600000
C	-0.05300000	0.40900000	0.48600000	H	-2.79700000	-5.07500000	4.60500000
H	-0.02000000	-0.29900000	1.32100000	C	-3.40800000	-3.31900000	1.79400000
C	0.21400000	1.92600000	2.53400000	H	-2.70300000	-2.57700000	1.41900000
H	0.23300000	2.95700000	2.89200000	C	-3.06400000	-4.78700000	1.40600000
H	1.15900000	1.45100000	2.80900000	H	-2.31900000	-4.94400000	0.63000000
H	-0.59500000	1.40200000	3.04700000	C	-5.82400000	2.85500000	-3.63000000
C	1.08500000	0.06300000	-0.47600000	O	-5.94000000	1.66100000	-3.63900000
H	1.11600000	0.79200000	-1.29300000	C	-6.86800000	3.85700000	-4.13300000
H	0.92400000	-0.91000000	-0.94600000	H	-7.79200000	3.70300000	-3.57500000
C	2.42700000	0.02300000	0.24400000	H	-7.07900000	3.64000000	-5.18100000
H	2.71100000	0.99700000	0.65200000	C	-6.26400000	5.25000000	-3.91900000
H	2.38100000	-0.66200000	1.09800000	H	-6.85700000	5.88200000	-3.25700000
C	3.54400000	-0.45500000	-0.65200000	H	-6.13900000	5.81300000	-4.84600000
O	3.40200000	-0.92200000	-1.74900000	C	-4.91000000	4.98900000	-3.29900000
O	4.73700000	-0.31500000	-0.05700000	C	-4.67700000	3.65800000	-3.14500000
C	5.86800000	-0.77200000	-0.82300000	C	-3.45000000	3.12200000	-2.57900000
H	5.90100000	-0.19700000	-1.75400000	O	-3.14000000	1.98800000	-2.41100000
H	5.71500000	-1.81700000	-1.09300000	O	-2.52600000	4.10600000	-2.19200000
C	7.08800000	-0.54000000	0.00500000	C	-3.93000000	5.95300000	-2.89100000
C	8.06400000	-1.41400000	0.25600000	C	-2.74500000	5.43100000	-2.32900000
H	7.16500000	0.46300000	0.41800000	C	-3.05100000	8.22100000	-2.58300000
C	8.09300000	-2.83200000	-0.25400000	C	-1.74500000	6.27400000	-1.89100000
H	8.73900000	-2.91400000	-1.13400000	H	-3.11700000	9.29500000	-2.66200000
H	7.10800000	-3.20300000	-0.53000000	C	-1.91200000	7.63800000	-2.03400000
H	8.50600000	-3.49600000	0.50900000	C	-4.05700000	7.36300000	-3.00800000
C	9.26300000	-1.00200000	1.07500000	O	-5.21100000	7.79700000	-3.55600000
H	9.11600000	0.01300000	1.45500000	C	-5.39900000	9.19400000	-3.70100000
H	9.34300000	-1.66400000	1.94700000	H	-5.38000000	9.69300000	-2.72900000
C	10.58000000	-1.06700000	0.29100000	H	-6.38000000	9.31500000	-4.15500000
H	10.49900000	-0.42800000	-0.59600000	H	-4.63600000	9.62500000	-4.35500000
H	10.73500000	-2.08900000	-0.06700000	O	-0.86500000	8.35400000	-1.57400000
C	11.77300000	-0.62600000	1.13800000	O	1.32900000	7.61300000	-1.74800000
H	11.54400000	0.34800000	1.58600000	C	0.63100000	5.52400000	-2.32800000
H	11.90200000	-1.32700000	1.97400000	H	0.59800000	4.56500000	-2.81900000
C	13.10000000	-0.51300000	0.37700000	C	1.51800000	6.48800000	-2.51700000
H	12.95500000	0.20200000	-0.44500000	H	2.38100000	6.53200000	-3.16400000
C	14.17900000	0.05600000	1.30700000	C	-0.38400000	6.00400000	-1.31500000
H	13.80600000	0.99100000	1.74100000	H	-0.41800000	5.38300000	-0.41800000
H	14.32300000	-0.63700000	2.14700000	C	0.12300000	7.45400000	-1.04700000
C	15.52700000	0.32700000	0.64000000	H	0.28900000	7.73700000	-0.01000000
H	15.36800000	0.91700000	-0.27300000				

chl a 2-E-2AFB$_1$

0 1	
Mg -2.50645900 -0.47135500 -0.88531700	H -0.64367300 1.62994100 3.89463700
N -3.40087500 -1.91114200 -1.99757600	C -1.47517200 -0.01772200 4.99614000
C -4.72343600 -2.24999900 -1.93675700	H -1.13066500 -1.00887800 5.32192700
C -4.95352800 -3.40813600 -2.80987500	H -1.44820200 0.61767100 5.88977600
C -3.75269500 -3.70992600 -3.38565100	C -2.92732400 -0.16331500 4.51619900
C -2.78793800 -2.74276500 -2.85264600	H -3.22836400 0.78178600 4.04070600
C -6.24847400 -4.06131000 -2.99602000	C -3.02786600 -1.28232200 3.46156700
H -7.12019600 -3.41152100 -3.01266000	H -2.59024900 -0.93721700 2.51891800
C -6.42454500 -5.37571600 -3.13000400	H -2.40163200 -2.12163700 3.79875500
H -7.41128700 -5.79870600 -3.27405600	C -4.43267500 -1.82502800 3.18963200
H -5.59026700 -6.06608300 -3.07365300	H -5.08020700 -1.01418000 2.83357800
C -3.43437900 -4.76282900 -4.39759900	H -4.87029600 -2.19444500 4.12529600
H -2.96309200 -5.63786200 -3.93723200	C -4.42726900 -2.97347200 2.17610000
H -2.74557700 -4.37661900 -5.15216200	H -4.28610100 -2.57569200 1.16578100
H -4.34243600 -5.09946100 -4.90011600	H -3.55444400 -3.60945100 2.37965900
C -5.68337500 -1.61855500 -1.17145500	C -5.67514800 -3.86257900 2.20893500
H -6.69251700 -2.00369200 -1.26446500	H -5.71709000 -4.34811400 3.19327900
N -4.31735100 0.00898500 0.04578200	C 2.09070400 2.42504900 3.83802700
C -5.50843600 -0.53401300 -0.26751700	H 2.81597900 2.97285100 3.23430000
C -4.55133300 1.02944900 0.93187300	H 2.39033400 2.49851900 4.88907000
C -5.97193000 1.11594500 1.20314300	H 1.13433300 2.94185700 3.72100400
C -6.57465000 0.14175100 0.45164600	C -3.83849500 -0.40773000 5.72158000
C -8.04378800 -0.14844000 0.38205500	H -3.69383700 0.36884100 6.47723300
H -8.28036700 -0.86078900 -0.40888600	H -3.60864800 -1.37318000 6.18644800
H -8.61108300 0.76364000 0.17933400	H -4.89542100 -0.41392800 5.44696900
H -8.41308000 -0.56702400 1.32288500	C -5.57134800 -4.94796600 1.13597100
C -6.62929200 2.04549600 2.17610400	H -5.48846700 -4.49008000 0.14320000
H -7.68591500 2.15335900 1.91555100	H -6.44995500 -5.59880300 1.13481900
H -6.18905300 3.04530300 2.10224300	H -4.68835100 -5.57483800 1.29270100
C -6.50540800 1.53418900 3.61793300	C -6.96327800 -3.06225900 2.02202800
H -6.99480000 2.20886200 4.32378400	H -6.96037100 -2.58016900 1.04145600
H -5.45464100 1.43703800 3.90134900	H -7.07021800 -2.28065600 2.77890200
H -6.96133700 0.54485300 3.71104900	H -7.84012700 -3.71335800 2.07850800
C -3.58654500 1.92550700 1.40053300	C -1.31241200 -3.08744700 0.31551500
H -3.93437400 2.70569700 2.07321100	O -1.94882200 -2.07217300 0.49524700
C -2.22925300 1.95008500 1.05736600	C -1.85387600 -4.42499900 -0.15437000
N -1.66207600 0.98897700 0.24075900	H -2.64939400 -4.29429900 -0.88702300
C -1.41734800 -2.72038400 -3.16515300	H -2.28886600 -4.91276100 0.72459600
H -1.07906800 -3.45986200 -3.88285700	C -0.61877200 -5.19087500 -0.65218100
C -0.43513400 -1.84223400 -2.69555500	H -0.54717500 -5.16847200 -1.74383300
N -0.59981800 -0.87182400 -1.79173700	H -0.58763800 -6.23718600 -0.34937000
C 0.58983500 -0.23526300 -1.54353800	C 0.54827400 -4.42785600 -0.07046200
C -0.39317400 1.32915400 0.09330300	C 0.13209600 -3.25468200 0.49157000
C 0.69475100 0.80408700 -0.65723200	C 1.01646300 -2.33278500 1.17811400
C -1.24866900 2.94402400 1.41843900	O 0.75722100 -1.27985200 1.67187800
C -0.08154100 2.52148400 0.80171000	O 2.32510500 -2.79434100 1.30513700
C -1.46644600 4.17163700 2.24204800	C 1.93343600 -4.77452500 -0.04969100
H -2.29031900 4.77232100 1.84009800	C 2.77773500 -3.90887900 0.68310400
H -0.56366200 4.78480300 2.24070600	C 3.88699300 -6.14127800 -0.62628800
H -1.71296600 3.91658500 3.27782400	C 4.12519100 -4.15284600 0.77409800
C 1.32334700 2.83750700 0.59212600	H 4.36310800 -6.97127300 -1.12501500
O 1.97442100 3.78721200 0.95798300	C 4.65595100 -5.25105900 0.12148900
C 1.90772700 1.61548000 -0.22105100	C 2.52194200 -5.89220500 -0.70119300
H 2.46055300 1.06116600 0.54316300	O 1.66585400 -6.66339800 -1.40113900
C 2.93887900 2.04203200 -1.23598700	C 2.19258100 -7.77857500 -2.10084100
O 4.04659200 2.46961800 -0.60680900	H 2.65685400 -8.48778000 -1.41031200
C 5.10736300 2.89481200 -1.46228400	H 2.92149200 -7.45751400 -2.84987500
H 5.52227300 2.03376300 -1.98845700	H 1.34445400 -8.24812200 -2.59229900
H 5.85523100 3.33012100 -0.80412800	O 5.98591000 -5.35451100 0.26541000
H 4.74781000 3.63084400 -2.18374500	O 7.13208100 -4.68405400 2.17117000
O 2.83946100 2.01160600 -2.43285900	C 5.26416500 -3.66649100 2.98397800
C 1.00189200 -1.91692700 -3.19849700	H 4.52331300 -3.32024500 3.68636900
H 1.42396900 -2.89417900 -2.92346700	C 6.34505600 -4.38445500 3.25493600
C 1.69854800 -0.78806700 -2.41276300	H 6.72126200 -4.75798500 4.19555500
H 1.99520700 0.01527700 -3.09368700	C 5.21868400 -3.42765800 1.49299800
C 1.09235300 -1.72752500 -4.71409500	H 5.23473400 -2.36873000 1.22940500
H 0.57742400 -2.52469400 -5.25400000	C 6.47056100 -4.22447600 1.01901000
H 2.13750600 -1.71637100 -5.03476600	H 7.17513200 -3.69956500 0.38155900
H 0.63723000 -0.77370000 -4.99139000	C -3.12717100 2.20248900 -2.22127900
C 2.92263500 -1.23481600 -1.60929300	O -3.10686900 1.01348000 -2.46722400
H 2.71237100 -2.19504200 -1.12877100	C -4.35268400 3.00822500 -1.82656100
H 3.13262700 -0.52193800 -0.80882300	H -4.99986500 2.41675300 -1.17787900
C 4.18395500 -1.36962500 -2.47348000	H -4.90499500 3.23309100 -2.74379600
H 4.11436700 -2.22010200 -3.15319600	C -3.79406000 4.28286800 -1.17722700
H 4.31452900 -0.44763400 -3.04699800	H -3.92779900 4.27261000 -0.09249300
C 5.38749700 -1.56733400 -1.58887600	H -4.24452500 5.20540600 -1.54413600
O 5.95577500 -2.61160500 -1.39009600	C -2.31731700 4.25202100 -1.49823300
O 5.73486100 -0.41700400 -0.99202700	C -1.97984200 3.10673800 -2.15635500
C 6.89044300 -0.45392500 -0.11820600	C -0.64786400 2.82759300 -2.66529100
H 7.74704200 -0.78332900 -0.71049000	O -0.31099800 1.94646000 -3.39098100
H 6.72254500 -1.19213500 0.66501800	O 0.31493800 3.75378200 -2.25665200
C 7.08418000 0.91895500 0.43277700	C -1.30491800 5.20458800 -1.16661200
C 6.59249000 1.36771900 1.59048600	C 0.01343300 4.87220100 -1.55570600
H 7.62780100 1.61061700 -0.20495000	C -0.45609700 7.23554600 -0.09159800
C 6.76697800 2.81070600 1.98066100	C 1.07003900 5.68599500 -1.21237800
H 5.78747500 3.29251600 2.07471600	H -0.57984600 8.14740300 0.47186600
H 7.35530900 3.36251800 1.24631000	C 0.81530600 6.84554400 -0.50586900
H 7.26095100 2.89173200 2.95313300	C -1.51039300 6.39534500 -0.42341700
C 5.77315300 0.55367200 2.56638800	O -2.78842800 6.63263900 -0.05133100
H 6.05524000 -0.50371400 2.55889900	C -3.04309200 7.76736500 0.75753100
H 5.98439200 0.92138000 3.57619300	H -2.48785200 7.70563200 1.69815200
C 4.26615700 0.70203600 2.29170800	H -4.11117200 7.75502900 0.95998900
H 4.03052500 0.11080100 1.39802900	H -2.78042800 8.68956400 0.23218500
H 4.06276300 1.74329500 2.02093300	O 1.92846000 7.55340700 -0.22723700
C 3.35766500 0.26942000 3.45262600	O 3.70832200 7.63812600 -1.72527900
H 3.22176300 -0.81685000 3.41469100	C 2.97271100 5.81630300 -2.88181300
H 3.84672700 0.49590700 4.40992100	H 2.76862900 5.14674200 -3.70216500
C 1.97862400 0.95191200 3.43366700	C 3.58154600 6.99236900 -2.93239000
H 1.58497900 0.88465800 2.41471300	H 4.01185700 7.52620200 -3.76638200
C 0.96034600 0.22943200 4.32405900	C 2.54677600 5.56971900 -1.45508600
H 1.09786100 -0.85280000 4.22738800	H 2.95681500 4.66135500 -1.01338400
H 1.15310800 0.48150800 5.37667600	C 3.06742400 6.85223200 -0.74605000
C -0.49502700 0.54631700 3.96583200	H 3.75298900 6.70643400 0.08572300

chl a 2-AFB$_1$c

0 1	
Mg -4.27683300 0.06861500 -1.14368300	H 6.03349300 3.25088200 -2.64852600
N -5.32605000 -1.64703700 -1.05747600	H 6.30728900 3.84132100 -1.00768900
C -6.64745500 -1.77465200 -0.68413400	C 4.38159700 2.12150200 0.20909900
C -7.02686900 -3.18491800 -0.77329300	H 4.60590900 1.26396400 0.84992200
C -5.90359600 -3.87263300 -1.13188000	H 4.70840000 3.01569900 0.75430000
C -4.85235200 -2.88469400 -1.31966000	C 2.86541700 2.18468700 -0.00906600
C -8.35500500 -3.75116200 -0.52669800	H 2.57865100 1.27765800 -0.55476500
H -8.38046900 -4.70687400 -0.00769800	H 2.62588100 3.02626400 -0.66892300
C -9.50403900 -3.19658700 -0.91149500	C 2.07566800 2.26749600 1.30068400
H -10.45235200 -3.67606900 -0.70223200	H 2.17716300 1.31022200 1.82664800
H -9.52602400 -2.26675900 -1.46873700	H 2.52058300 3.02951900 1.95496200
C -5.73888600 -5.34278700 -1.33924000	C 0.58835300 2.59412000 1.09648000
H -4.98667000 -5.75148500 -0.65803800	H 0.21863500 1.97227900 0.27003300
H -5.41482200 -5.56027900 -2.36062500	C -0.28686600 2.24625600 2.30779400
H -6.67668500 -5.87122000 -1.16803900	H 0.11484500 1.36898500 2.82787200
C -7.42636100 -0.75600500 -0.18225000	H -0.25714300 3.07406200 3.02822400
H -8.41603700 -1.02940100 0.16536500	C -1.73834200 1.95157500 1.91174800
N -5.89563400 1.14021200 -0.45439100	H -1.76671600 0.97680400 1.40977500
C -7.06437800 0.60565900 -0.03579600	H -2.08630900 2.68046200 1.16929400
C -5.95271200 2.48602300 -0.17759800	C -2.68227500 1.97322500 3.11230600
C -7.20681400 2.79672200 0.46959900	H -2.21476600 1.43970200 3.95207600
C -7.89955100 1.61702800 0.56695300	H -2.79235700 3.01366300 3.44067700
C -9.23942400 1.36663000 1.18494800	C -4.07297000 1.36544000 2.88516000
H -9.16885100 0.62652300 1.98717900	H -4.52610400 1.83516300 2.00105100
H -9.95498700 0.98735200 0.44984800	C -3.96710200 -0.15106700 2.62829800
H -9.65152700 2.28146200 1.61169500	H -3.61484200 -0.33212500 1.60224300
C -7.58630300 4.14251500 1.00641700	H -3.18536800 -0.56244000 3.28230700
H -8.67411900 4.21868200 1.07485800	C -5.24872700 -0.95016000 2.85957400
H -7.27069600 4.92429500 0.30957100	H -6.07171700 -0.50032400 2.29175500
C -6.96108400 4.39286000 2.38538200	H -5.52419500 -0.87903500 3.91811100
H -7.21481100 5.38564700 2.76252300	C -5.10013500 -2.42705900 2.48954300
H -5.87229000 4.30757100 2.33316800	H -5.15885800 -2.54154000 1.40075600
H -7.31421700 3.64849700 3.10267600	H -4.10015200 -2.77260300 2.78086400
C -4.94937500 3.40885000 -0.48308700	C -6.13558000 -3.34586800 3.15093700
H -5.15539100 4.45198700 -0.26316400	H -5.93165800 -3.35038700 4.22972400
C -3.70664800 3.11612600 -1.04071500	C 0.40645800 4.07161500 0.73214400
N -3.30578000 1.81136400 -1.29593300	H 0.92066800 4.34692900 -0.18994700
C -3.54184300 -3.19016100 -1.71473400	H 0.80440600 4.69387200 1.54028700
H -3.31943500 -4.24122200 -1.85572800	H -0.64926900 4.32199700 0.60477100
C -2.49914200 -2.30540400 -1.95620100	C -4.96031300 1.67962300 4.09289500
N -2.54521900 -0.96532900 -1.83179500	H -4.96284300 2.75324700 4.29557800
C -1.33088000 -0.41373600 -2.18086200	H -4.58952800 1.17116800 4.98987900
C -2.07211400 1.89727700 -1.77314300	H -5.99431400 1.36831600 3.93004700
C -1.10578500 0.93684700 -2.16461100	C -5.98697100 -4.77387100 2.62653400
C -2.65999200 4.03927400 -1.40405500	H -6.21902100 -4.80185400 1.55630700
C -1.63228900 3.23939100 -1.87451000	H -6.66479900 -5.46062900 3.13998800
C -2.68869000 5.52724900 -1.27685100	H -4.96583900 -5.14207100 2.75734400
H -3.49416800 5.95821300 -1.87691400	C -7.56750400 -2.85012500 2.94409700
H -1.74257400 5.94748500 -1.61718000	H -7.79086200 -2.76980800 1.87709000
H -2.85045500 5.83010400 -0.23820800	H -7.72579900 -1.86755100 3.39495000
C -0.27068100 3.24408700 -2.41366400	H -8.28680000 -3.54371500 3.38775800
O 0.43991100 4.15824600 -2.73675500	C 8.02168000 3.02008000 1.07179700
C 0.16278700 1.72873200 -2.44041500	O 8.61539600 3.73918200 0.31410900
H 0.84436900 1.66473500 -1.58931900	C 7.34307300 3.46697600 2.36978000
C 0.99861500 1.36457500 -3.64094400	H 6.63913200 4.26777300 2.13477400
O 2.24256300 1.81575100 -3.46724300	H 8.10214600 3.88594400 3.03236500
C 3.17049600 1.51445800 -4.50998100	C 6.66254200 2.22111000 2.94791000
H 3.38837100 0.44508000 -4.50888800	H 5.57829600 2.32280500 3.02677300
H 4.06783800 2.07993500 -4.27400600	H 7.01655000 1.95541900 3.94550300
H 2.76361800 1.80974700 -5.47680200	C 6.99963000 1.11563900 1.97420300
O 0.63446600 0.73398200 -4.59778500	C 7.76748500 1.56909200 0.94614300
C -1.12901700 -2.78966600 -2.40815900	C 8.21304900 0.72277600 -0.14884000
H -0.68653100 -3.36790300 -1.58718700	O 8.84731300 1.02081100 -1.10823100
C -0.34518700 -1.47938600 -2.61100000	O 7.82842600 -0.62187300 -0.02259100
H -0.13317700 -1.32434400 -3.67488400	C 6.57010800 -0.25041600 2.01505300
C -1.18197700 -3.66598800 -3.66032200	C 7.02433000 -1.08021700 0.96460700
H -1.73315300 -4.59120600 -3.48408400	C 5.25399000 -2.12232900 2.89292900
H -0.17105300 -3.93114400 -3.97945400	C 6.60908000 -2.39163600 0.87313300
H -1.66792100 -3.12844400 -4.47779100	H 4.56933700 -2.56706300 3.59846400
C 0.97576900 -1.41199300 -1.84553900	C 5.73821000 -2.88173300 1.83077400
H 0.79574500 -1.61225000 -0.78296600	C 5.68336600 -0.80459200 2.97686700
H 1.39780500 -0.40931500 -1.91237400	O 5.27785300 0.03932700 3.94934400
C 2.02669000 -2.38478000 -2.39501900	C 4.36817000 -0.44785500 4.92060500
H 1.76123100 -3.42704200 -2.21471800	H 3.43261600 -0.76712900 4.45301600
H 2.13052700 -2.22112800 -3.47265100	H 4.17573200 0.38537500 5.59172300
C 3.36131100 -2.11968100 -1.74305600	H 4.80504300 -1.27891500 5.48087500
O 3.96867900 -2.88951200 -1.04154800	O 5.37743000 -4.15799400 1.62163200
O 3.77851700 -0.87797200 -2.01190500	O 6.75560200 -5.74823400 0.64159800
C 4.96776100 -0.45431600 -1.30272300	C 8.26299100 -4.13676000 0.07888800
H 5.75962900 -1.17544300 -1.51099700	H 9.20920100 -3.65065300 -0.09468700
H 4.74285800 -0.49573100 -0.23518100	C 8.07115600 -5.37330000 0.51431300
C 5.35571700 0.90854500 -1.76474000	H 8.78070600 -6.14656000 0.76754000
C 5.13202700 2.04655300 -1.10151400	C 6.91136300 -3.48663900 -0.10934000
H 5.88151900 0.95301300 -2.71446800	H 6.70831900 -3.19648500 -1.14137400
C 5.57368700 3.36688200 -1.66673300	C 5.96287000 -4.61531600 0.38831300
H 4.71819500 4.04490000 -1.75367400	H 5.14324500 -4.88813900 -0.26847900

Reference

1.- Politzer, P.; Murray, J.S. The fundamental nature and role of the electrostatic potential in atoms and molecules. *Theor Chim Acta* **2002**, *108*,134-142.
